# Sulforaphane Exposure Prevents Cadmium-Induced Toxicity and Mitochondrial Dysfunction in the Nematode *Caenorhabditis elegans* by Regulating the Insulin/Insulin-like Growth Factor Signaling (IIS) Pathway

**DOI:** 10.3390/antiox13050584

**Published:** 2024-05-09

**Authors:** Estefani Yaquelin Hernández-Cruz, Omar Emiliano Aparicio-Trejo, Dianelena Eugenio-Pérez, Elí Juárez-Peredo, Mariana Zurita-León, Víctor Julián Valdés, José Pedraza-Chaverri

**Affiliations:** 1 Laboratorio F-315, Departamento de Biología, Facultad de Química, Universidad Nacional Autónoma de México (UNAM), Mexico City 04510, Mexico; estefani.hernandez@quimica.unam.mx (E.Y.H.-C.); dian.eugenio@comunidad.unam.mx (D.E.-P.); 316298434@quimica.unam.mx (E.J.-P.); 2 Posgrado en Ciencias Biológicas, Universidad Nacional Autónoma de México (UNAM), Ciudad Universitaria, Mexico City 04510, Mexico; 3 Departamento de Fisiopatología Cardio-Renal, Instituto Nacional de Cardiología “Ignacio Chávez”, Mexico City 14080, Mexico; omar.aparicio@cardiologia.org.mx; 4 Posgrado en Ciencias Bioquímicas, Universidad Nacional Autónoma de México (UNAM), Biochemical Sciences, Ciudad Universitaria, Mexico City 04510, Mexico; 5 Departamento de Biología y Desarrollo Celular, Instituto de Fisiología Celular (IFC), Universidad Nacional Autónoma de México (UNAM), Mexico City 04510, Mexico; mzurita@ifc.unam.mx (M.Z.-L.); julian.valdes@ifc.unam.mx (V.J.V.)

**Keywords:** *Caenorhabditis elegans*, sulforaphane, cadmium toxicity, mitochondrial, oxidative stress

## Abstract

Cadmium (Cd) is a heavy metal that is highly toxic to humans and animals. Its adverse effects have been widely associated with mitochondrial alterations. However, there are not many treatments that target mitochondria. This study aimed to evaluate the impact of sulforaphane (SFN) pre-exposure against cadmium chloride (CdCl_2_)-induced toxicity and mitochondrial alterations in the nematode *Caenorhabditis elegans* (*C. elegans*), by exploring the role of the insulin/insulin-like growth factor signaling pathway (IIS). The results revealed that prior exposure to SFN protected against CdCl_2_-induced mortality and increased lifespan, body length, and mobility while reducing lipofuscin levels. Furthermore, SFN prevented mitochondrial alterations by increasing mitochondrial membrane potential (Δψm) and restoring mitochondrial oxygen consumption rate, thereby decreasing mitochondrial reactive oxygen species (ROS) production. The improvement in mitochondrial function was associated with increased mitochondrial mass and the involvement of the *daf-16* and *skn-1c* genes of the IIS signaling pathway. In conclusion, exposure to SFN before exposure to CdCl_2_ mitigates toxic effects and mitochondrial alterations, possibly by increasing mitochondrial mass, which may be related to the regulation of the IIS pathway. These discoveries open new possibilities for developing therapies to reduce the damage caused by Cd toxicity and oxidative stress in biological systems, highlighting antioxidants with mitochondrial action as promising tools.

## 1. Introduction

Cadmium (Cd) is a highly toxic heavy metal with no biological function. It is primarily found in its divalent form (Cd^2+^) and is constantly released into the environment from anthropogenic and natural sources [1,2]. Exposure can occur either occupationally or environmentally. Workers in electroplating, battery production, and pigment industries are at the highest risk of Cd exposure [3]. In addition, environmental exposure occurs through air pollution, consumption of contaminated food and water, and smoking [4,5]. Attempts have been made to control exposure sources; however, Cd pollution remains a significant issue in many developing countries due to its accumulation [6].

Cd has a long half-life and bioaccumulates in plants, invertebrates, and vertebrates [5]. Toxic effects following exposure include growth retardation and toxicity in different body systems [5,7]. It causes obstructive respiratory diseases, emphysema, end-stage renal failure, diabetes, blood pressure disorders, bone diseases, immunosuppression, premature aging, cancer, and even death [8,9,10]. Numerous studies have shown that mitochondria play a fundamental role in Cd toxicity [11,12]. Cd enters mitochondria and affects the electron transport system (ETS), increasing the reactive oxygen species (ROS) production and decreasing the mitochondrial membrane potential (Δψm), which induces alterations in mitochondrial dynamics, mutations in mitochondrial deoxyribonucleic acid (mtDNA), and decreases in mitochondrial biogenesis [11,12,13,14]. Currently, there is no specific or effective treatment for Cd poisoning [7]. Furthermore, among the few treatments available, none is targeted to treat mitochondrial dysfunction.

Conversely, sulforaphane (SFN) is an isothiocyanate derived from the hydrolysis of glucosinolates found in cruciferous vegetables such as broccoli, cauliflower, and cabbage [15]. In recent years, SFN has garnered interest in mitochondrial target studies due to growing evidence suggesting that SFN decreases alterations in mitochondrial dynamics, Δψm, and bioenergetics [16]. The main SFN mechanism of action is the activation and nuclear translocation of the nuclear factor erythroid 2-related factor 2 (Nrf2), which stimulates the transcription of the enzymatic antioxidant battery [17,18]. Previously, SFN has been observed to protect against Cd toxicity in different organs and cell types, including the liver [19], testes [20], Leydig cells (TM3) [21], Sertoli cells [22], mesenchymal stem cells [23], and peripheral blood lymphocytes and monocytes [24]. However, whether the SFN protective effects on Cd-induced damage are related to mitochondrial protection is still unclear. Furthermore, the molecular mechanism has not yet been elucidated.

Finally, the nematode *Caenorhabditis elegans* (*C. elegans*) stands as a valuable and extensively utilized model for investigating the impact of phytochemicals on mitochondrial function and the toxicity of environmental chemicals [25,26]. The use of this animal has helped the discovery of several conserved signaling pathways involved in Cd detoxification mechanisms [27,28]. Notably, it has also been found that human genes responsible for various mitochondrial diseases have orthologous genes in this nematode, making it possible to use *C. elegans* as a model organism to study different mitochondrial alterations [29]. Similarly, oxidative stress signaling pathways are highly conserved, especially the insulin/insulin-like growth factor signaling (IIS) pathway [30,31]. Hence, it would be of predictive value to study the mechanisms of action of pro-oxidant agents, such as Cd, and antioxidants, such as SFN, in this model. This study aimed to investigate the protective potential of SFN against Cd-induced toxicity and mitochondrial alterations using the nematode *C. elegans* as a model. Additionally, the role of the IIS pathway in this interaction was analyzed to better understand the underlying mechanisms of SFN action in this process.

## 2. Materials and Methods

### 2.1. Reagents

Sulforaphane (SFN-S8044) was purchased from LKT Laboratories, Inc. (St Paul, MN, USA). Cadmium chloride (CdCl_2_-C-2544), cholesterol (C3045), yeast extract (70161), 5-fluoro-2′-desoxiuridina (FUdR-F0503), dimethyl sulfoxide (DMSO), streptomycin sulfate salt (S6501), sodium azide (S2002), antimycin A (A8674), and rotenone (R8875) were purchased from Sigma-Aldrich (St. Louis, MO, USA). Sodium hypochlorite (NaClO) was purchased from Cloralex (Oakland, CA, USA). 12% Parmisole (Levamisole HCl 12 g-Q-0021-006) was purchased from PARFAM, S.A. (Ciudad de México, México). MitoTracker^TM^ Green FM (M7514), MitoSOX^TM^ Red mitochondrial superoxide indicator (M36008), 5,5,6,6′-tetrachloro-1,1′,3,3′ tetraethylbenzimi-dazoylcarbocyanine iodide (JC-1-T3168), 6-chloromethyl-2′,7′-dichlorodihydrofluorescein diacetate, acetyl ester (CM-H_2_DCFDA-C6827), bacto-agar (214010), bacto^TM^ peptone, and bacto^TM^ tryptone were purchased from Thermo Fisher Scientific (Waltham, MA, USA). Magnesium sulfate heptahydrate (MgSO_4_•7H_2_O-2500-01), potassium phosphate monobasic (KH_2_PO_4_-3246-01), sodium hydrogen phosphate (Na_2_HPO_4_-3828-01), sodium hydroxide (NaOH-46697-2002), and sodium chloride (NaCl-7647-14-5) were purchased from JT Baker (Xalostoc, Edo. Mex., Mexico). Potassium phosphate dibasic (K_2_HPO_4_-7088) and potassium chloride (KCl-6858) were purchased from Mallinckrodt, AR (St. Louis, MO, USA).

### 2.2. C. elegans Strains

The following *C. elegans* strains used in this study were purchased from the *Caenorhabditis* Genetics Center (University of Minnesota, Minneapolis, MN, USA), including N2 (wild-type, Bristol) [32], VC128 *mtl-2* (gk125) *V* [33], RB1623 *cdr-2* (ok1996) V [33], TK22 *mev-1* (kn1) III [34,35], TJ1052 *age-1*(hx546) II [33,36], QV225 *skn-1* (zj15) *IV* [37], GR2245 *skn-1* (mg570) IV [38,39], GR1307 *daf-16(mgDf50)I* [35], and the reporter strain SJ4143 zcIs17 (Pges-1::GFPmt) [40,41], QQ202 *daf-2*(cv20[*daf-2*::GFP]) III, and OH16024 *daf-16*(ot971[*daf-16*::GFP]) I [42]. The characteristics of the strains are summarized in Table S1.

### 2.3. C. elegans Culture

*C. elegans* was cultured in Nematode Growth Medium complete (NGM: 0.3% NaCl, 1.7% agar, 2.5% peptone, 0.1% potassium phosphate buffer (1 M, pH 6), 5 µg/mL cholesterol, 1 mM CaCl_2_, and 1 mM MgSO_4_) on plastic plates seeded with a lawn of *E. coli* OP50-1 bacteria (CGC, University of Minnesota, Minneapolis, MN, USA). *E. coli* OP50-1 bacteria were cultured overnight at 37 °C in liquid Lysogeny broth (LB: 10 g bacto^TM^ tryptone, 5 g yeast extract, 5 g sodium chloride, and 1000 mL ddH_2_O) and diluted to an OD_600_ of approximately 0.3, as previously described [43]. Subsequently, 1000 µL of the *E. coli* OP50-1 suspension was seeded onto 60 mm NGM plates and dried overnight at room temperature. The worms were maintained according to previous standard protocols [44] with an incubation temperature of 20 °C.

### 2.4. Experimental Design

**Figure 1:**
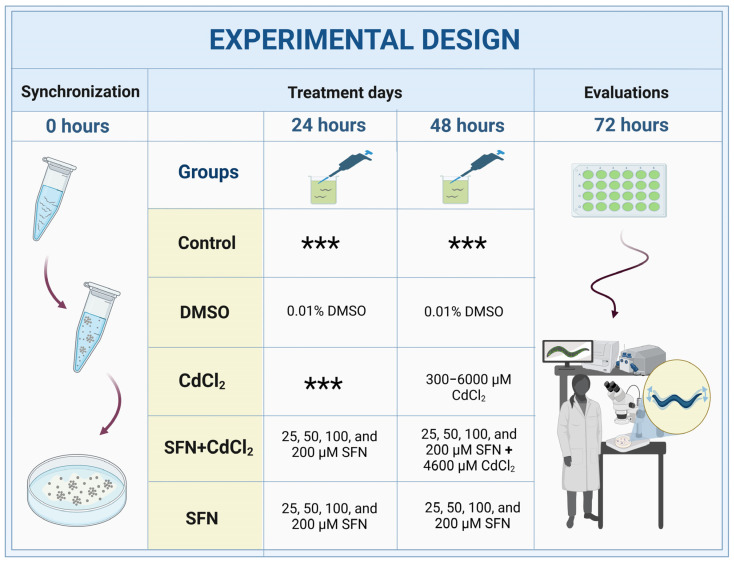
Experimental design with *C. elegans*. DMSO: dimethyl sulfoxide, CdCl_2_: cadmium chloride, SFN: sulforaphane. *** indicates that nematodes are cultured only with K medium and OP50-1 bacteria (1:10 dilution).

*C. elegans* were collected from NGM plates using M9 buffer (6 g Na_2_HPO_4_, 3 g KH_2_PO_4_, 5 g NaCl, 0.25 g MgSO_4_•7H_2_O, and 1000 mL ddH_2_O), and cultures were synchronized using a bleaching solution (NaOH 0.5 M and 0.5% NaClO) [45]. Subsequently, the eggs were allowed to hatch in NGM boxes with *E. coli* OP50-1 bacteria. Twenty-four hours after synchronization, worms in the L1 stage were transferred to 24-well plates with K medium (52 mM NaCl and 32 mM KCl), OP50-1 bacteria (1:10 dilution), and the different treatments. There were five experimental groups: (I) Control, without treatment; (II) DMSO, 48 h with 0.1% DMSO; (III) CdCl_2_, 24 h with the different concentrations of CdCl_2_ (300–6000 μM); (IV) SFN+CdCl_2_, 24 h with SFN (25, 50, 100, and 200 μM) plus 24 h with SFN and CdCl_2_ (4600 μM); and (V) SFN, 48 h with the different concentrations of SFN (25, 50, 100, and 200 μM) (Figure 1). All evaluations were carried out at the end of the exposures when most of the worms were in the adult stage. To prepare the SFN concentrations, a 100 mM stock solution was prepared in DMSO and diluted 1:1000 in K medium. For CdCl_2_ concentrations, a 1 M stock solution was prepared in milliQ water and then diluted to 50 mM in K medium [46,47]. Note: To evaluate mitochondria-associated oxygen consumption, the exposures to the treatments were not carried out in a liquid medium (review Section 2.10).

### 2.5. Survival

Nematodes from the N2, VC128, RB1623, TK22, TJ1052, GR1307, GR2245, and QV225 strains were exposed to the different treatments in 24-well clear plates at 20 °C (30 ± 2 nematodes per well). After exposure, nematodes were classified, under a dissecting microscope, as either alive or dead. Dead nematodes did not move after a gentle touch and showed no pharyngeal pumping [48]. Three to five independent assays were conducted, with three replicates per concentration in each experiment. Concentration-response curves were obtained for N2 worms exposed to CdCl_2_.

### 2.6. Lifespan Analysis

The lifespan assays were conducted as described by Takahashi et al. [49]. Following exposure to the different treatments, random samples of 20 N2 nematodes were transferred to NGM plates containing 25 μM FUdR and *E. coli* OP50-1 and incubated at 20 °C. Day 0 was considered the day in which worms were transferred to NGM plates. Worms that no longer responded to gentle touch with a platinum wire were classified as dead and removed from the plates. The endpoint was considered the day when all worms were dead. At least five independent experiments were conducted. Finally, the Kaplan–Meier method was used to calculate the survival percentage using GraphPad Prism software version 10.

### 2.7. Lipofuscin Assay

Lipofuscin is a senescence marker in nematodes that can be analyzed using fluorescence microscopy [50,51]. After exposure to the different treatments, random samples of 20 N2 nematodes were placed in black, clear-bottomed 96-well plates. Nematodes were paralyzed with 5 mM Levamisole, and images were taken with the Cytation™ 5 using a blue fluorescence filter to DAPI. Images were analyzed using Fiji 1.54f (See Section 2.14) [52]. At least three independent experiments were conducted.

### 2.8. Body Length

After exposure to the different treatments, random samples of 20 N2 nematodes were placed in black, clear-bottom 96-well plates. Nematodes were paralyzed with 5 mM Levamisole, and images were taken with the Cytation™ 5. Subsequently, body length was measured using Fiji 1.54f [52]. Three independent experiments were conducted.

### 2.9. Mobility/Body-Bending Assay

Body bends are used to reflect worm mobility [47]. After exposure to the different treatments, random samples of 10 N2 nematodes per group were transferred to freshly prepared NGM plates without food and left at 20 °C for 2 min. Subsequently, they were individually examined for body bends, defined as a change in the direction of the posterior bulb or regular head oscillation. Body bends were counted for 60 s. Three independent experiments were conducted.

### 2.10. Mitochondria-Associated Oxygen Consumption

Mitochondrial respiration measurement was conducted as described by Branicky et al. [53] with slight modifications. Briefly, N2 nematodes were exposed to different treatments: (I) control, (II) DMSO (0.01%), (III) CdCl_2_ (4600 μM), (IV) SFN (100 μM) + CdCl_2_ (4600 μM), and (V) SFN (100 μM), on NGM plates with *E. coli* OP50-1 bacteria. The DMSO, SFN, and CdCl_2_ were prepared in OP50-1 bacteria DO_600_ = 0.3. Subsequently, 2 mL was placed on the 100 mm × 15 mm NGM plates and allowed to dry for 24 h before placing the worms. After exposure to the different treatments, the worms were washed with M9 buffer and loaded into the 2 mL chamber of the Oroboros Oxygraph 2K. The number of worms was counted in 3 aliquots of 20 µL of the worm suspension to determine the total number of worms in the chamber. Basal respiration was measured after N2 nematode addition at 20 °C, and residual respiration (ROX) was obtained by the titration with 25 µL of 2M sodium azide, 10 µL of 5 mM antimycin, plus 5 µL of 1 mM rotenone. The ROX value was subtracted from the basal respiration to determine the value of the mitochondrial respiration rate. The mitochondrial respiration rate was normalized to the number of worms in the chamber, which averaged around 1000 nematodes. Three independent experiments were conducted.

### 2.11. Use of Fluorescent Probes

Fluorescent probes were used to assess mitochondrial mass, Δψm, and intracellular and mitochondrial ROS (Table 1). N2 nematodes were exposed to the different treatments according to the scheme in Figure 1. However, before completing the exposure time, the corresponding probe was added to the K medium, considering the incubation time of each of the probes (Example: the H_2_DCFDA probe was added 4 h before completing treatments). This was done to ensure simultaneous completion of treatment exposure and probe incubation. Subsequently, nematodes were washed with M9 buffer to remove excess dye and then plated on NGM plates with *E. coli* bacteria for 1 h at 20 °C to remove excess probe from the intestine. Nematodes were paralyzed with 5 mM Levamisole in black, clear-bottom 96-well plates. Images were taken with the Cytation™ 5 and analyzed using Fiji 1.54f (See Section 2.14) [52]. The MitoSOX^TM^ Red/MitoTracker^®^ Green fluorescence ratio was determined for mitochondrial ROS. At least three independent experiments were conducted.

### 2.12. Quantification of Green Fluorescent Proteins (GFP)

After exposure to the different treatments, random samples of nematodes from the SJ4143 and QQ202 strains were taken and placed in black, clear-bottom 96-well plates. Nematodes were paralyzed with 5 mM Levamisole, and images were taken with the Cytation™ 5 using a green fluorescence filter. Images were analyzed using Fiji 1.54f (See Section 2.14) [52]. At least three independent experiments were conducted.

### 2.13. Nuclear Localization of DAF-16

The nuclear localization of DAF-16 was measured as described previously [54]. After exposure to the different treatments, random samples of 15 nematodes from the transgenic strain OH16024, expressing a DAF-16::GFP fusion protein, were taken and placed in black, clear-bottom 96-well plates. Nematodes were paralyzed with 5 mM Levamisole, and images were taken with the Cytation™ 5 using a green fluorescence filter. The nuclear localization of DAF-16::GFP was classified as cytosolic, intermediate, and nuclear, as proposed by Oh et al. [55]. At least three independent experiments were conducted.

### 2.14. Image Analyses

Image analyses were performed in Fiji 1.54f [52]. A macro for executing the following procedures was written. We segmented each worm by automatically thresholding the 8-bit image and manually selecting the contours of the worms. Each contour was added to the region of interest (ROI) manager, and the fluorescence intensity for each ROI was determined in the channel of interest. Background fluorescence for each image was also determined by adding random background rectangles to the ROI manager. Data were extracted, the background subtracted for each worm, and the fluorescence normalized to the control group.

### 2.15. Statistical Analysis and Calculation of the Mean Lethal Concentration (LC_50_)

The results are presented as mean ± standard error of the mean (SEM). Normality and homoscedasticity of variances were assessed using the Shapiro–Wilk and Bartlett tests, respectively. Differences between groups were determined using one-way analysis of variance (ANOVA) followed by Tukey’s test. If normality was not met, the Kruskal–Wallis test was used, followed by Dunn’s test. When comparing the two groups, an unpaired *t*-test was performed. Data from lifespan analysis were analyzed using Kaplan–Meier analysis and a log-rank test. Statistical analyses were conducted using GraphPad Prism 10™ software (GraphPad Software, Inc., San Diego, CA, USA). Differences were considered significant at *p* ≤ 0.05. To calculate the LC_50_, two different analyses were performed: a simple logistic regression with GraphPad Prism 10™ and a Probit analysis with Miller–Tainter corrections. Finally, for nuclear translocation of DAF-16, a multinomial logistic regression model was fitted, treatment effect analyzed through a chi-square test, and pairwise comparisons made using Tukey's method (R 4.3.0 software). 

## 3. Results

### 3.1. SFN Prevents CdCl_2_-Induced Decrease in Survival

**Figure 2:**
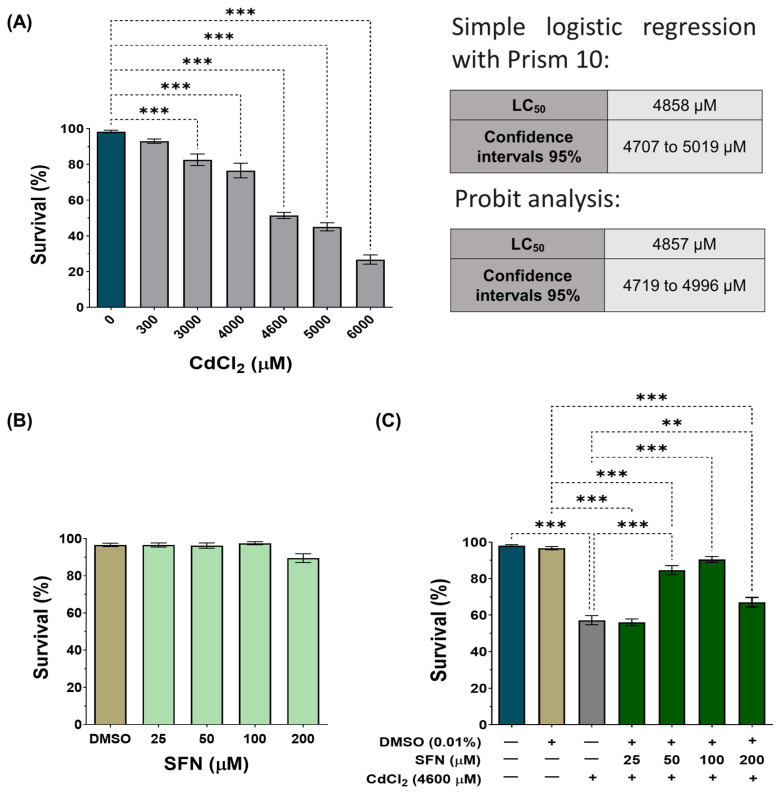
Effect of sulforaphane (SFN) pre-exposure on the survival of *Caenorhabditis elegans* (*C. elegans*) exposed to cadmium chloride (CdCl_2_). Bristol N2 nematodes in the L1 stage were exposed to different concentrations of (**A**) CdCl_2_ for 24 h, (**B**) SFN for 48 h, and (**C**) SFN with 4600 μM of CdCl_2_ (24 h of SFN alone plus 24 h of SFN+CdCl_2_). After the treatments, nematodes were scored under a dissecting microscope as alive if they were moving or as dead if they did not respond to gentle probing. Percentage calculations were made based on the obtained data. Data are presented as mean ± SEM, n = 3 to 5 independent bioassays with 3 technical replicates each. ** *p* < 0.01, *** *p* < 0.001.

To validate our model, wild-type *C. elegans* (Bristol-N2) were exposed to various concentrations of CdCl_2_ (300–6000 μM). A concentration-dependent effect was observed, with the concentration of 6000 μM decreasing survival by 73%, while at the concentration of 300 μM, the decrease was only 7% (Figure 2A). To determine the LC_50_, we performed two different analyses: a simple logistic regression with GraphPad Prism 10™ and a Probit analysis that showed an LC_50_ of 4858 μM (IC95: 4707–4019 μM) and 4857 μM (IC95: 4719–4996 μM), respectively (Figure 2A and Supplementary Figure S1). These concentrations are similar to each other and to those previously reported by other authors [46,56]. Subsequently, nematodes were exposed to concentrations of SFN between 25 and 200 μM to demonstrate that SFN alone had no toxic effects. As expected, SFN had no significant effect on the survival of *C. elegans* (Figure 2B). Finally, we evaluated whether pre-exposure to SFN prevents the death of nematodes caused by exposure to 4600 μM of CdCl_2_ (a concentration close to the lower limit of the LC_50_ of CdCl_2_ and the one chosen to carry out the remaining experiments). To do this, the nematodes were exposed for 24 h to different concentrations of SFN, followed by another 24 h of coincubation with SFN and CdCl_2_. Exposure to 50 and 100 μM SFN increased survival by 27.45 and 34.01%, respectively, compared to the group treated only with CdCl_2_. It should be noted that pre-exposure of 100 μM of SFN achieved a survival rate of 90.5% (Figure 2C). Since protection was highest at 100 μM, subsequent experiments were performed using only this concentration of SFN. These data demonstrate that pre-exposure of SFN prevents CdCl_2_-induced death in *C. elegans*.

### 3.2. SFN Prevents the Toxic Effects Induced by CdCl_2_

**Figure 3:**
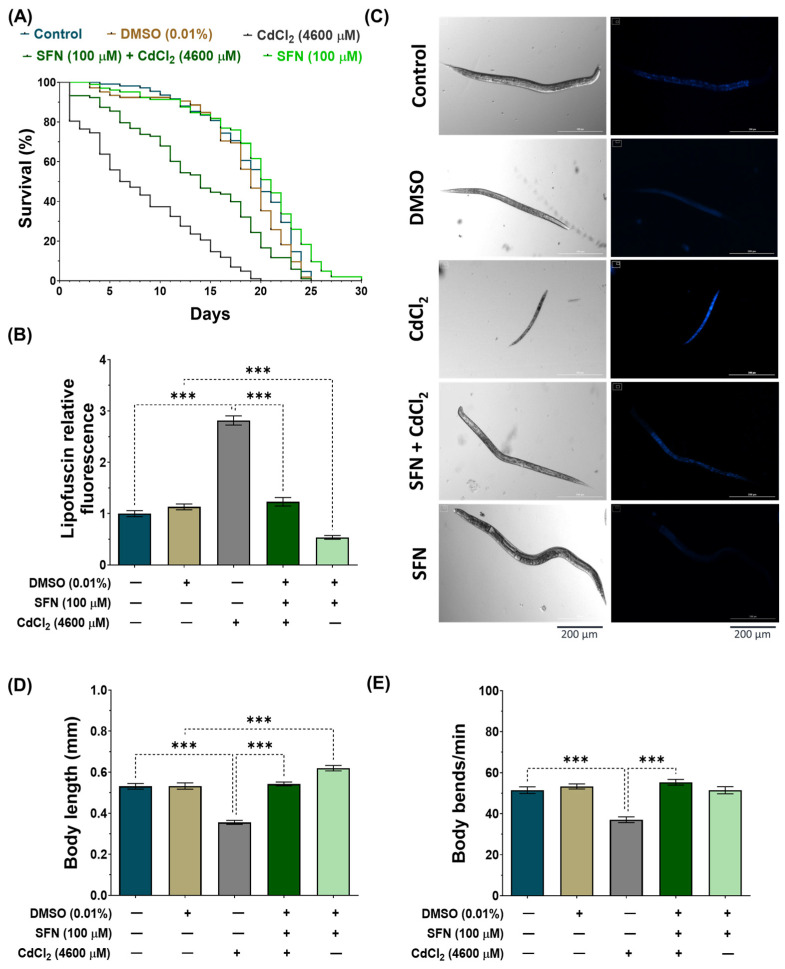
Protective effect of sulforaphane (SFN) exposure against cadmium chloride (CdCl_2_)-induced toxicity in *Caenorhabditis elegans* (*C. elegans*). Bristol N2 nematodes at the L1 stage were initially exposed to SFN. After 24 h, they were re-exposed, this time to a combination of SFN and CdCl_2_ for another 24 h. Additional groups were created and exposed to 0.1% dimethyl sulfoxide (DMSO, SFN vehicle) for 48 h, CdCl_2_ (4600 μM) for 24 h, and SFN (100 μM) for 48 h. Once the exposure times were over, the corresponding evaluations were carried out. (**A**) Kaplan–Meier curves for lifespan (n ≥ 100 nematodes divided into 5 independent experiments), (**B**) lipofuscin levels corrected for background in each image and normalized to the control group (n = 60 nematodes distributed in 3 independent experiments), (**C**) representative images of lipofuscin levels and body size, (**D**) body size (n = 60 nematodes distributed in 3 independent experiments), and (**E**) body bends (n = 30 nematodes distributed in 3 independent experiments). Data are presented as mean ± SEM, *** *p* < 0.001.

To evaluate whether SFN pre-exposure mitigates the toxic effects of CdCl_2_ by increasing the lifespan and improving the health of *C. elegans*, we analyzed the mean and maximum lifespan, lipofuscin levels, body size, and body bends of the nematodes. In the lifespan evaluation (Figure 3A and Table 2), it was found that exposure to CdCl_2_ drastically decreased the lifespan to a mean of 8 ± 0.56 days (maximum lifespan of 20 days) compared to the control group, which was 19 ± 0.45 days (maximum lifespan of 25 days). Meanwhile, in the SFN+CdCl_2_ group, the decrease in nematode lifespan was prevented, as the mean lifespan was 14 ± 0.68 days (maximum lifespan of 25 days), corresponding to a 42.9% increase compared to the group treated with CdCl_2_ alone. Furthermore, and consistent with previous reports [47], exposure to SFN alone increased the mean lifespan of *C. elegans* to 20 ± 0.58 days (maximum lifespan of 30 days) compared to the DMSO group. On the other hand, lipofuscin accumulation levels were evaluated (Figure 3B,C), a pigment associated with aging or cellular deterioration. Lipofuscin is a complex mixture of oxidized protein and lipid degradation residues, along with minor amounts of carbohydrates and metals. It originates from an incomplete lysosomal digestion of the phagocyte and self-phagocyte material, for example, from incomplete lysosomal degradation of damaged mitochondria [57,58]. In addition, it has been observed that additional oxidative stress promotes its formation [59]. In this study, we observed a significant increase in lipofuscin levels in the CdCl_2_-exposed group compared to the control group, indicating a physiological aging state possibly associated with increased oxidative stress. However, in the SFN+CdCl_2_-exposure group, this increase was prevented by 21.1%, suggesting a protective effect of SFN against Cd-induced lipofuscin accumulation. Furthermore, exposure to SFN alone reduced lipofuscin levels by 52.51% compared to the DMSO group. Other toxicity parameters evaluated were body size (Figure 3C,D) and body bends (Figure 3E). We found that the nematodes in the CdCl_2_-exposed group were smaller, and the number of body bends per minute decreased significantly compared with the control group. Meanwhile, in the SFN+CdCl_2_ group, nematodes statistically increased their body length and bends by 34.5% and 33%, respectively, compared to the group exposed to CdCl_2_ alone. Finally, the SFN-exposed group only increased body size compared to the DMSO group and did not affect body bends. Overall, these results indicate that pre-exposure of SFN prevents toxic effects and increases the lifespan of *C. elegans* exposed to CdCl_2_.

**Figure 4:**
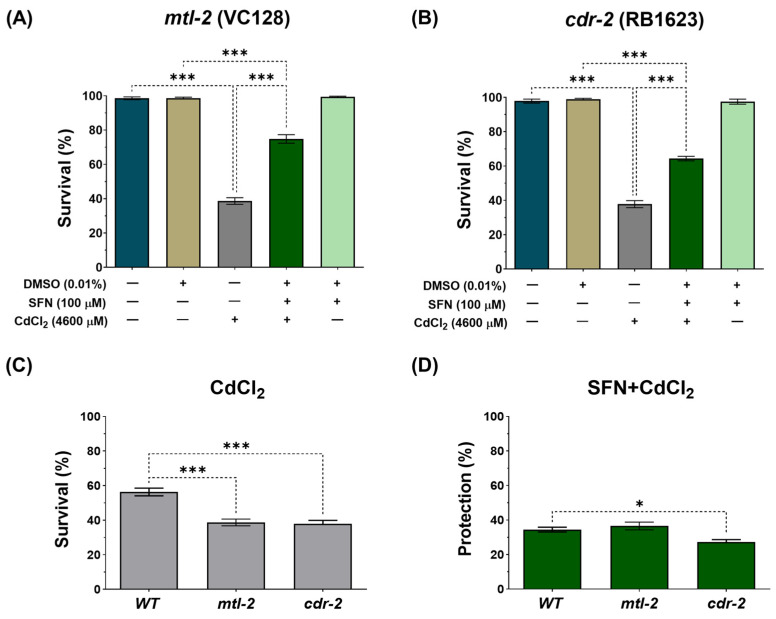
Effect of pre-exposure to sulforaphane (SFN) on response genes to cadmium chloride (CdCl_2_) toxicity in *Caenorhabditis elegans* (*C. elegans*). Nematodes of strains VC128 (mtl-2 mutant) and RB1623 (cdr-2 mutant) in stage L1 were initially exposed to SFN. After 24 h, they were exposed again, this time to a combination of SFN and CdCl_2_ for another 24 h. Additional groups were created and exposed to 0.1% dimethyl sulfoxide (DMSO, SFN vehicle) for 48 h, CdCl_2_ (4600 μM) for 24 h, and SFN (100 μM) for 48 h. Once the exposure times were completed, survival was evaluated. (**A**) Survival percentage of strain VC128 (*mtl-2* mutant), (**B**) Survival percentage of strain RB1623 (*cdr-2* mutant), (**C**) Effect of CdCl_2_ on the survival of mutant strains compared to the wild type (WT), and (**D**) Percent survival protection in the SFN+CdCl_2_ mutant strain group compared to WT. *mtl-2*: metallothionein 2, *cdr-2*: cadmium response gene 2. Data are presented as mean ± SEM, n = 3 independent bioassays with 3 technical replicates each. * *p* < 0.05, *** *p* < 0.001.

It has been reported that in response to Cd, *C. elegans* upregulates several hundred genes, including two metallothioneins, 1 and 2 (*mtl-1* and *mtl-2*), and the Cd-responsive genes, *cdr-1* to *cdr-7* [60,61]. To assess whether these genes play a role in the protection conferred by SFN against CdCl_2_ toxicity, survival was evaluated in the mutant strains VC128 and RB1623, which have mutated genes *mtl-2* and *cdr-2*, respectively. It was observed that SFN pre-exposure protects from death induced by CdCl_2_ exposure in both strains (Figure 4A,B). However, deleting these genes made *C. elegans* more hypersensitive to CdCl_2_ toxicity (Figure 4C). Furthermore, protection in the RB1623 strain decreased by 7.49% compared to the protection observed in the wild-type (WT) strain (Figure 4D). These data suggest that the *cdr-2* gene might be associated with SFN protection against Cd toxicity.

### 3.3. SFN Prevents CdCl_2_-Induced Mitochondrial Dysfunction

**Figure 5:**
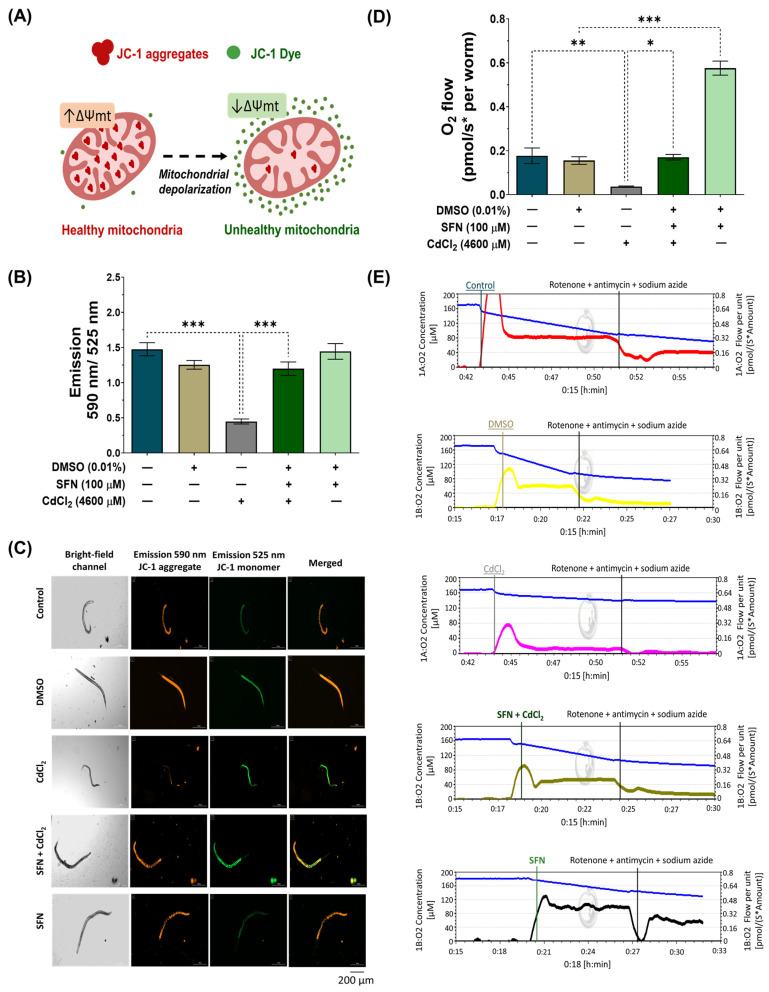
Effect of sulforaphane (SFN) pre-exposure on mitochondrial dysfunction in *Caenorhabditis elegans* (*C. elegans*) exposed to cadmium chloride (CdCl_2_). Bristol N2 nematodes at the L1 stage were initially exposed to SFN. After 24 h, they were exposed again, this time to a combination of SFN and CdCl_2_ for another 24 h. Additional groups were created and exposed to 0.1% dimethyl sulfoxide (DMSO, SFN vehicle) for 48 h, CdCl_2_ (4600 μM) for 24 h, and SFN (100 μM) for 48 h. Once the exposure times were completed, the corresponding evaluations were carried out. (**A**) Schematic illustration depicting the entry of 5,5′,6,6′-tetrachloro-1,1′,3,3′-tetraethylbenzimidazolylcarbocyanine iodide (JC-1) into mitochondria and the generation of J-aggregates; JC-1, a cationic carbocyanine dye (green), accumulates in mitochondria in a potential-dependent manner, where it forms J aggregates (red), and upon depolarization, it remains as a monomer showing green fluorescence, (**B**) Evaluation of Δψm with JC-1 probe by calculating the fluorescence ratio at 590 nm/525 nm, (**C**) Representative images of JC-1 probe fluorescence (scale bar indicates 200 μM), (**D**) Quantification of oxygen consumption (basal oxygen consumption minus oxygen consumption in the presence of electron transport system inhibitors), and (**E**) Representative graphs of oxygen consumption measurement by Oroboros Oxygraph 2K. n = 45 nematodes distributed in 3 independent experiments. Data are presented as mean ± SEM, * *p* < 0.05, ** *p* < 0.01, *** *p* < 0.001.

Cd directly affects mitochondrial homeostasis [11]. Thus, to assess whether SFN prevents CdCl_2_-induced mitochondrial dysfunction, Δψm was evaluated using the JC-1 probe, and mitochondrial-associated oxygen consumption rate was evaluated by high-resolution respirometry. For Δψm measurements, nematodes were exposed to the different treatments together with the JC-1 probe, followed by capturing images with red and green fluorescence. JC-1 is a lipophilic, cationic dye that can selectively enter mitochondria and reversibly change color from green to red with increasing membrane potential. In polarized mitochondria, JC-1 spontaneously forms complexes known as J-aggregates with intense red fluorescence. In contrast, JC-1 remains in the monomeric form in depolarized mitochondria, exhibiting only green fluorescence (Figure 5A). Our results demonstrate that in the group exposed to CdCl_2_, Δψm decreases by 69.67% compared to the control group. However, exposure to SFN prevents the decrease in Δψm, as there was a 62.65% increase compared to the group exposed to CdCl_2_, which is statistically significant. Finally, the group exposed only to SFN showed no significant effects (Figure 5B,C).

ΔΨm is a key indicator of mitochondrial activity, as it reflects the process of electron transport that determines the oxidative phosphorylation as a driving force behind adenosine triphosphate (ATP) production [62]. Therefore, the reduction in mitochondrial respiration rate triggers lower ΔΨm, favoring the depletion in ATP production. The mitochondrial oxygen-consumption rate was evaluated to determine if SFN preserves ΔΨm by ETS maintaining. It was found that exposure to CdCl_2_ decreased mitochondrial oxygen consumption by 79.11% compared to the control group. Similarly to the ΔΨm assessment, the SFN+CdCl_2_ group preserved mitochondrial function, showing an increase of 78.29% compared to the CdCl_2_-exposed group. Interestingly, it was observed that the group treated only with SFN increased the mitochondrial oxygen-consumption rate above control values (Figure 5D,E).

### 3.4. SFN Prevents the Decrease in Mitochondrial Mass Induced by CdCl_2_

**Figure 6:**
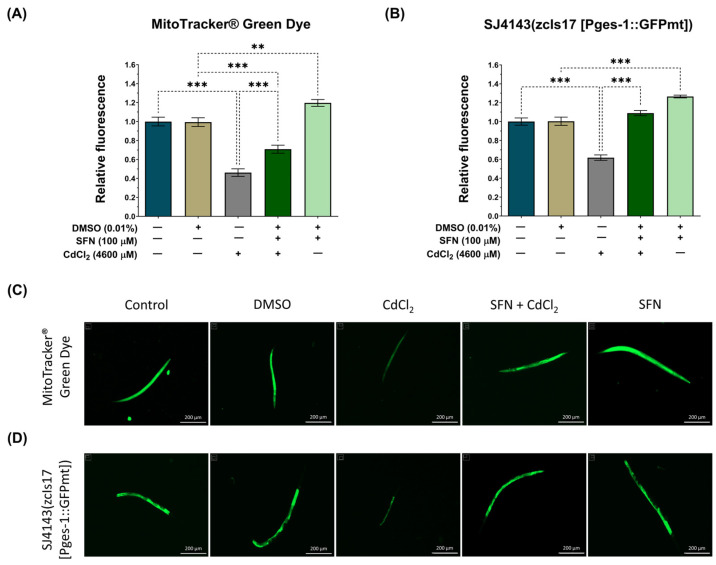
Effect of sulforaphane (SFN) pre-exposition on mitochondrial mass in *Caenorhabditis elegans* (*C. elegans*) exposed to cadmium chloride (CdCl_2_). Bristol N2 nematodes and SJ4143 zcIs17 (Pges-1::GFPmt) nematodes at the L1 stage were initially exposed to SFN. After 24 h, they were exposed again, this time to a combination of SFN and CdCl_2_ for another 24 h. Additional groups were created and exposed to 0.1% dimethyl sulfoxide (DMSO, SFN vehicle) for 48 h, CdCl_2_ (4600 μM) for 24 h, and SFN (100 μM) for 48 h. Once the exposure times were completed, the nematodes were paralyzed with levamisole, and images were taken using the Cytation™ 5. The fluorescence intensity was evaluated using Fiji 1.54f. (**A**) Relative fluorescence of the MitoTracker^®^ Green probe (n = 36 nematodes divided into 3 independent experiments), (**B**) Relative fluorescence of the strain SJ4143 zcIs17 (Pges-1::GFPmt) (n = 80 nematodes divided into four independent experiments), (**C**,**D**) Representative fluorescence images of the MitoTracker^®^ Green probe and the SJ4143 zcIs17 (Pges-1::GFPmt) strain, respectively (scale bar indicates 200 μM). GFP: green fluorescent protein, mt: mitochondria. Data are presented as mean ± SEM, ** *p* < 0.01, *** *p* < 0.001.

It has been demonstrated that SFN increases mitochondrial mass in HHL-5 cells and rats fed a high-fat diet, enhancing ETS function and increasing ΔΨm [63]. Therefore, to elucidate whether the preservation of mitochondrial bioenergetics by SFN in nematodes exposed to CdCl_2_ was associated with increased mitochondrial mass, we utilized the MitoTracker^®^ Green probe to assess the mitochondrial mass of the entire worm. Additionally, considering that the intestine is the primary organ exposed to this metal, where more significant damage would be expected [64], we employed the transgenic reporter strain SJ4143 zcIs17(Pges-1::GFPmt) to evaluate mitochondrial mass in the worm’s intestine. Our results show that exposure to CdCl_2_ decreases total and intestinal mitochondrial mass compared to the control group. Meanwhile, in the SFN+CdCl_2_ group, preservation of mitochondrial mass was observed. In the evaluation with the MitoTracker^®^ Green probe, an increase of 24.39% was observed (Figure 6A,C), and in the measurement with the SJ4143 strain, an increase of 44.04% was observed (Figure 6B,D) compared to the CdCl_2_ group. These results suggest that one of the mechanisms by which SFN prevents mitochondrial dysfunction is by increasing mitochondrial mass. Interestingly, in the group treated only with SFN, a statistically significant increase in mitochondrial mass compared to the DMSO group was observed. This can explain the increase in the mitochondrial oxygen-consumption rate reported in Figure 5D, where a higher number of mitochondria leads to higher oxygen consumption. With the MitoTracker^®^ Green probe, the increase was 19%, and with the SJ414 strain, the increase was 26%.

### 3.5. SFN Prevents Oxidative Damage Induced by CdCl_2_

**Figure 7:**
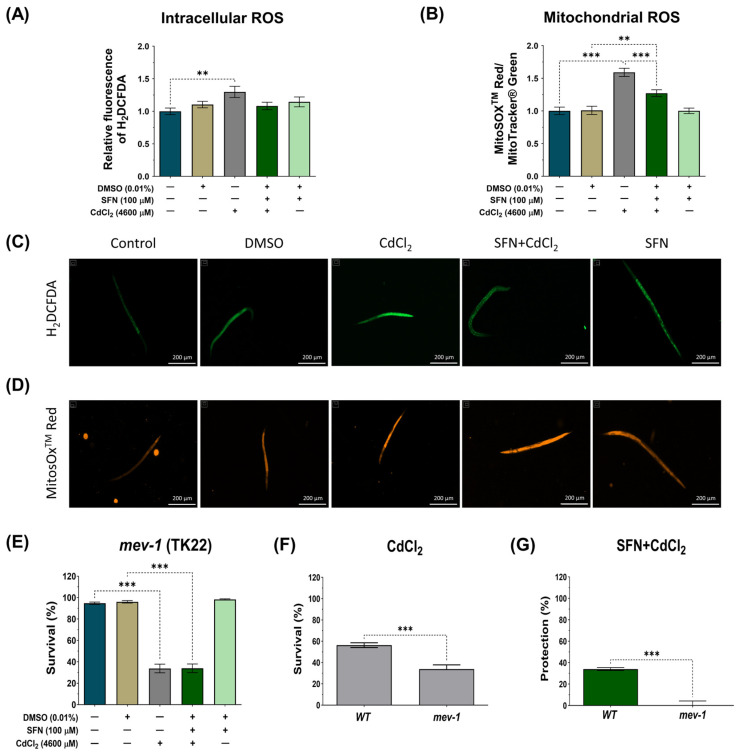
Protective effect of sulforaphane (SFN) pre-exposure against oxidative damage induced by cadmium chloride (CdCl_2_) in *Caenorhabditis elegans* (*C. elegans*). Bristol N2 and nematodes of strain TK22 (*mev-1* mutants) were initially exposed to SFN. After 24 h, they were exposed again, this time to a combination of SFN and CdCl_2_ for another 24 h. Additional groups were created and exposed to 0.1% dimethyl sulfoxide (DMSO, SFN vehicle), CdCl_2_ (4600 μM) for 24 h, and SFN (100 μM) for 48 h. Once the exposure times were completed, the corresponding evaluations were carried out. (**A**) Relative fluorescence of the 2′,7′-dichlorofluorescein diacetate (H_2_DCFDA) probe representing intracellular reactive oxygen species (ROS) (n= 45 nematodes divided into 3 independent experiments), (**B**) Ratio of MitoSOX^TM^ Red/MitoTracker^®^ Green to determine the Mitochondrial ROS (n = 36 nematodes divided into 3 independent experiments), (**C**,**D**) Representative images of the fluorescence of the H_2_DCFDA and MitoSOX^TM^ Red probes, respectively (scale bar indicates 200 μM), (**E**) Percent survival of strain TK22 (*mev-1* mutant) (n = three independent bioassays with 3 technical replicates each), (**F**) Effect of CdCl_2_ on strain survival of strain TK22 (*mev-1* mutant) compared to wild strain (WT) represented in percentage, and (**G**) Percentage of survival protection in the SFN+CdCl_2_ group of strain TK22 (*mev-1* mutant) compared to WT. Data are presented as mean ± SEM, ** *p* < 0.01, *** *p* < 0.001.

The Cd toxicity is primarily manifested as an increase in oxidative stress [65]. Mitochondrial dysfunction is one of the main mechanisms by which Cd increases ROS levels, thereby exacerbating mitochondrial alterations and generating a vicious cycle [11]. The effect of SFN on intracellular and mitochondrial ROS levels was evaluated to determine if protecting mitochondrial function could prevent CdCl_2_-induced ROS production. N2 nematodes and the probes H_2_DCFDA and MitoSOX^TM^ Red were used to assess intracellular and mitochondrial ROS levels, respectively. Mitochondrial ROS levels were calculated by the ratio of MitoSOX^TM^ Red probe fluorescence to MitoTracker^®^ Green probe fluorescence to ensure that the number of mitochondria did not mask the effects on ROS levels. In the case of intracellular ROS, CdCl_2_ was found to significantly increase ROS by 10.4% compared to the control group. However, although there was a trend towards decreased intracellular ROS levels in the SFN+CdCl_2_ group, these data were not statistically significant (Figure 7A,C). On the contrary, when mitochondrial ROS were evaluated, it was found that CdCl_2_ increased levels by 59.2% compared to the control group. In the SFN+CdCl_2_ group, mitochondrial ROS levels decreased by 20.11% compared to those exposed to CdCl_2_ alone (Figure 7B,D). These data suggest that SFN prevents ROS production, mainly in the mitochondria.

On the other hand, in *C. elegans*, *mev-1* encodes a subunit of succinate-coenzyme Q oxidoreductase in complex II of the electron transport chain [66,67]. Mutation of *mev-1* increases superoxide levels and hypersensitivity to oxygen, indicating that *mev-1* may modulate the cellular response to oxidative stress [66,67]. It has also been observed that *mev-1* governs the rate of aging by modulating the cellular response to oxidative stress in *C. elegans* [66]. Therefore, we investigated survival in the TK22 strain with a loss-of-function mutation in *mev-1* exposed to CdCl_2_ and evaluated the effect of SFN on it to determine its protective capacity against oxidative damage. As shown in Figure 7F, the *mev-1* mutation resulted in hypersensitivity to CdCl_2_; however, this effect was not attenuated with SFN treatment, as indicated in Figure 7E,G. These data suggest that the protective effect of SFN against CdCl_2_-induced oxidative damage may occur through a *mev-1*-dependent pathway in nematodes.

### 3.6. SFN-Mediated Protection against CdCl_2_ Toxicity Is Mediated by the IIS Pathway

**Figure 8:**
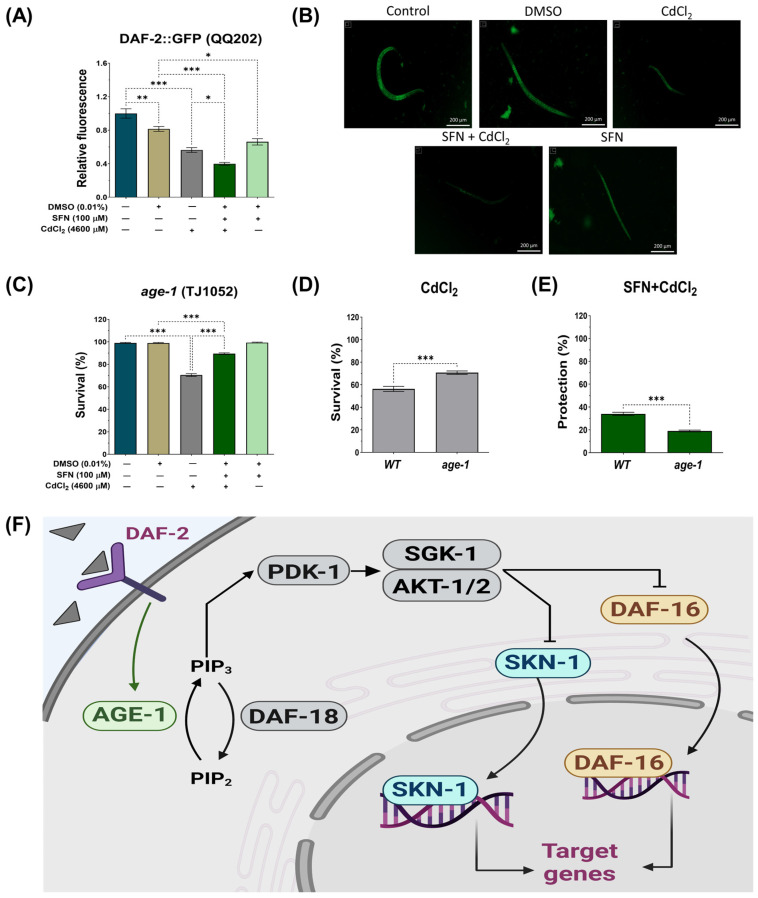
Effect of sulforaphane (SFN) pre-exposure on the insulin/insulin-like growth factor (IIS) signaling pathway in *Caenorhabditis elegans* (*C. elegans*) exposed to cadmium chloride (CdCl_2_). Nematodes of strains QQ202 (daf-2 reporter) and TJ1052 (*age-1* mutant) at the L1 stage were initially exposed to SFN. After 24 h, they were exposed again, this time to a combination of SFN and CdCl_2_ for another 24 h. Additional groups were created and exposed to 0.1% dimethyl sulfoxide (DMSO, SFN vehicle) for 48 h, CdCl_2_ (4600 μM) for 24 h, and SFN (100 μM) for 48 h. Once the exposure times were completed, the corresponding evaluations were carried out. (**A**) Relative fluorescence of DAF-2::GFP (n = 60 nematodes divided into 4 independent experiments), (**B**) representative fluorescence images of strain QQ202 (*daf-2*(cv20[*daf-2*::GFP]) (scale bar indicates 200 μM), III), (**C**) percent survival of strain TJ1052 (*age-1* mutant), (**D**) effect of CdCl_2_ on the survival of strain TJ1052 (*age-1* mutant) compared to wild type (WT), (**E**) percentage of survival protection in the SFN+CdCl_2_ group of strain TJ1052 (*age-1* mutant) compared to WT (n= 3 independent bioassays with 3 technical replicates each), and (**F**) schematic representation of signaling the IIS path. *age-1*: phosphatidylinositol 3-kinase orthologous gene, AGE-1: phosphatidylinositol 3-kinase gene protein, AKT1-2: protein kinases B, *daf-16*: forkhead box O (FoxO) orthologous gene, DAF-18: PTEN/human tumor suppressor homolog, *daf-2*: a receptor tyrosine kinase (IGFR) homolog, DAF-2: receptor tyrosine kinase (IGFR) homologous gene protein, PDK-1: 3-phosphoinositide-dependent protein kinase 1, PIP3: phosphatidylinositol 3,4,5-trisphosphate, SGK-1: glucocorticoid-regulated kinase 1, SKN-1c: ortholog of nuclear factor erythroid 2-related factor 2 (Nrf2). Data are presented as mean ± SEM, * *p* < 0.05, ** *p* < 0.01, *** *p* < 0.001.

The IIS pathway in *C. elegans* regulates various biological processes, including metabolism, growth, development, longevity, stress response, and behavior [31]. This essential pathway operates through the interaction of insulin-like peptide ligands with the transmembrane receptor DAF-2, an analog of the insulin/insulin-like growth factor-1 (IGF-1) receptor (IGFR). Activation of DAF-2/IGFR triggers a series of events, including activation of phosphatidylinositol 3-kinase (AGE-1), which generates phosphatidylinositol 3,4,5-trisphosphate (PIP3). In turn, PIP3 activates 3-phosphoinositide-dependent protein kinase 1 (PDK-1), which phosphorylates and activates protein kinases B (AKT1-2) and glucocorticoid- and serum-regulated kinase 1 (SGK-1). This complex process culminates in regulating transcription factors such as DAF-16, which controls most of the functions of this pathway, and skn-1 [31] (Figure 8F). To determine whether the IIS pathway is involved in protecting SFN against Cd toxicity, the expression of DAF-2 was determined using strain QQ202 (*daf-2* reporter) and the survival of worms from strain TJ1052 (*age-1* mutant). It was observed that exclusive exposure to CdCl_2_ caused a 44% reduction in DAF-2 expression compared to the control group. However, this decrease was even more pronounced in the SFN+CdCl_2_ group, where a 50.72% reduction was recorded compared to the DMSO group and 32.82% compared to the group exposed only to CdCl_2_ (Figure 8A,B). These findings indicate that pre-exposure of SFN to CdCl_2_ exerts a synergistic effect in reducing DAF-2 expression.

On the other hand, when evaluating the survival of the *age-1* mutant, it was observed that pre-exposure to SFN continued to protect against CdCl_2_-induced death. Although this protection was decreased compared to the WT strain, survival still reached almost 90% (Figure 8C,E). Notably, in worms exposed exclusively to CdCl_2_, survival increased compared to the WT strain, suggesting that *age-1* mutants are more resistant to CdCl_2_ (Figure 8D).

**Figure 9:**
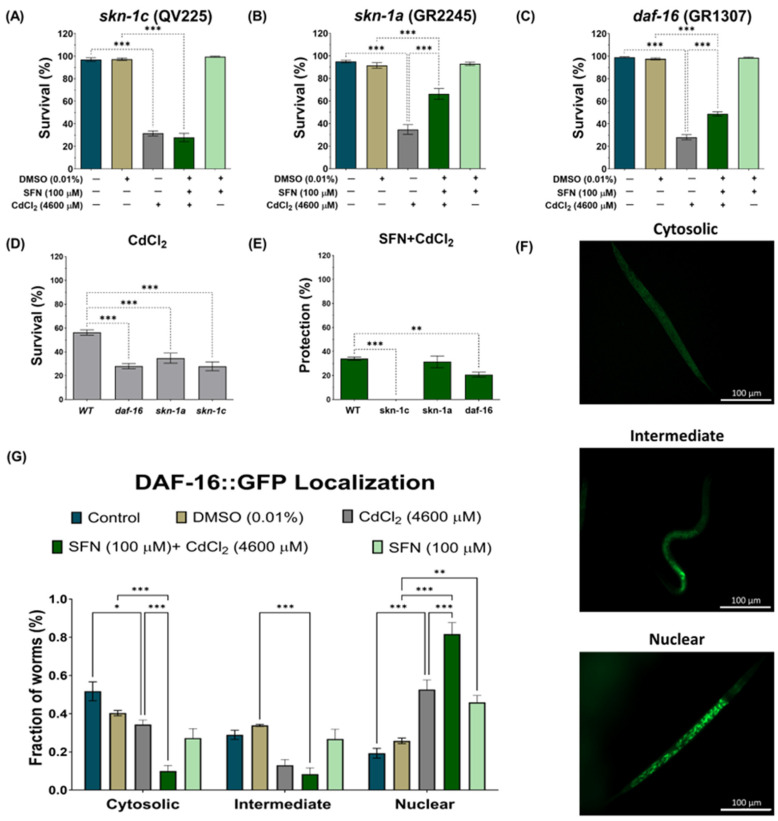
Exploration of the participation of *skn-1* and *daf-16* in the protection of sulforaphane (SFN) pre-exposure against cadmium chloride (CdCl_2_) toxicity in *Caenorhabditis elegans* (*C. elegans*). Nematodes of strains QV225 (*skn-1c* mutant), GR2245 (*skn-1a* mutant), GR1307 (*daf-16* mutant), and OH16024 (*daf-16* reporter) at the L1 stage were initially exposed to SFN. After 24 h, they were exposed again, this time to a combination of SFN and CdCl_2_ for another 24 h. Additional groups were created and exposed to 0.1% dimethyl sulfoxide (DMSO, SFN vehicle) for 48 h, CdCl_2_ (4600 μM) for 24 h, and SFN (100 μM) for 48 h. Once the exposure times were completed, the corresponding evaluations were carried out. (**A**) Percentage survival of strain QV225 (*skn-1c* mutant), (**B**) percentage survival of strain GR2245 (*skn-1a* mutant), (**C**) percentage survival of strain GR1307 (*daf-16* mutant), (**D**) effect of CdCl_2_ on the survival of mutant strains compared to wild type (WT), (**E**) percentage of survival protection in the SFN+CdCl_2_ mutant strain group compared to WT, (**F**) representative images of the cytosolic, intermediate, and nuclear categories, used to evaluate the nuclear translocation of DAF-16 and (**G**) nuclear localization of DAF-16 presented as the fraction of nematodes classified in each category (cytosolic, intermediate and nuclear). n of the survival assays = 3 independent bioassays with 3 technical replicates, and n of the nuclear translocation assay = 60 nematodes divided into three independent experiments. daf-16: forkhead box O orthologous gene (FoxO), DAF-16: FoxO orthologous gene protein, GFP: green fluorescent proteins, skn-1c: nuclear factor erythroid-related 2 (Nrf2) ortholog, skn1a: ortholog of Nrf1. Data are presented as mean ± SEM, * *p* < 0.05, ** *p* < 0.01, *** *p* < 0.001.

To further elucidate our findings, we examined whether SFN could target the transcription factor DAF-16/FOXO and SKN-1/Nrf downstream of DAF-2. SKN-1/Nrf proteins are members of the cap ‘n’ collar (CNC) family of transcription factors, which act as master regulators of oxidative stress resistance and longevity [68]. The *skn-1* gene encodes four major predicted isoforms, of which, three are expressed in vivo (*skn-1a* to *skn-1c*) [69]. In particular, the *skn-1c* isoform has been described as the ortholog of Nrf2 in mammals, one of the main targets of SFN [17]. Furthermore, *skn-1c* has been reported to be important for mitochondrial biogenesis and health in *C. elegans* [70]. To determine whether *skn-1c* plays a role in protecting SFN against CdCl_2_ toxicity, survival was assessed in strain QV225 (*skn-1c* mutant). As expected, deletion of *skn-1c* caused nematodes to be hypersensitive to CdCl_2_ toxicity (Figure 9D). Furthermore, it was observed that SFN lost its ability to prevent the decline in survival in nematodes exposed to CdCl_2_, as protection decreased from 34.01% in WT to −3.604% in *skn-1c* mutants (Figure 9A,E). These results indicate that *skn-1c* is crucial in protecting SFN against CdCl_2_ toxicity. Subsequently, we evaluated whether the *skn-1a* isoform could also be involved in the protection of SFN against CdCl_2_ toxicity. *skn-1a* is the ortholog of Nrf1 in mammals and has been detected in preparations of endoplasmic reticulum (ER) and mitochondria. Its main functions have been associated with the ER stress response and support of proteostasis [69]. This evaluation was included because Cd has been shown to alter protein folding in the ER and induce an unfolded protein response [71], which has also been associated with mitochondrial alterations [72]. However, our results showed that *skn-1a* is unrelated to SFN protection, as similar protection is still observed in strain GR2245 (*skn-1a* mutant) compared to the WT strain (Figure 9B,E). In particular, nematodes were more sensitive to CdCl_2_ toxicity in the mutant strain than in the WT (Figure 9D). Another gene involved in resistance to oxidative stress is *daf-16*, a human homolog of FoxO [73,74]. It was recently shown that SFN increased the nuclear translocation of DAF-16, thereby increasing the longevity of *C. elegans* [47]. Furthermore, DAF-16 is important in responding to Cd toxicity [28]. Therefore, to determine the role of DAF-16 in protecting SFN against CdCl_2_ toxicity, the survival of the mutant strain GR1307 was evaluated. We found that *daf-16* deletion nematodes showed increased sensitivity to CdCl_2_ toxicity (Figure 9D), while exposure to SFN prior to CdCl_2_ increased survival by 19.61% compared to exclusive exposure to CdCl_2_ (Figure 9C). Despite this, the protection offered is significantly lower than that observed in the WT strain (Figure 9E). Additionally, we determined the nuclear translocation of DAF-16 using strain OH16024 with GFP markers. The nuclear localization of DAF-16::GFP was classified as cytosolic, intermediate, or nuclear, as proposed by Oh et al. [55] (Figure 9F). Exposure to CdCl_2_ alone was found to increase DAF-16 nuclear translocation compared to the control group; however, in the SFN+CdCl_2_ group, the increase in nuclear translocation was much more significant compared to the CdCl_2_ group (Figure 9G). Together, these findings point out that regulation of the IIS pathway is critical for SFN-mediated protection against CdCl_2_ toxicity in *C. elegans*, particularly in the activation of DAF-16/FOXO and SKN-1c/Nrf2.

## 4. Discussion

The purpose of this study was to examine whether exposure to SFN can influence the toxicity and mitochondrial dysfunction induced by Cd in the nematode *C. elegans*, as well as to explore any possible involvement of the IIS pathway in this process. Our results revealed that prior exposure to SFN prevents the toxic effects induced by CdCl_2_, since it was observed that the SFN+CdCl_2_ group maintained survival above 90%. Furthermore, there was a significant increase in lifespan, body length, and mobility, as well as a decrease in lipofuscin levels, compared to the group exposed exclusively to CdCl_2_ (Figure 2 and Figure 3). Although previous studies had demonstrated the ability of SFN to mitigate Cd cytotoxicity [19,20,21,22,23,24], this is the first study to demonstrate a similar effect in the nematode *C. elegans*.

It is currently known that mitochondrial dysfunction is key in Cd toxicity, and previous studies have shown that SFN can improve mitochondrial malfunction [75,76]. However, there is limited information on whether SFN specifically prevents Cd-induced mitochondrial dysfunction. Exposure to Cd triggers oxidative stress, damaging mitochondria and affecting their energy efficiency, which impacts cellular health and organism functioning. Various studies indicate that Cd also directly affects mitochondrial function, altering internal metabolic processes [11,14]. Cd enters mitochondria through the voltage-dependent anion channel (VDAC), divalent metal transporter 1 (DMT1), and mitochondrial calcium uniporter (MCU) [77,78]. Once inside, it can disrupt the electron transport system, increase ROS production, and dissipate ΔΨm, triggering mitochondrial uncoupling [11,13]. Additionally, it reduces enzymatic activity in the Krebs cycle, damages mitochondrial DNA, and decreases mitochondrial biogenesis [79,80]. The Cd also affects the expression of key genes such as sirtuin 1 (Sirt1), nuclear respiratory factors 1 (NRF1), peroxisome proliferator-activated receptor-gamma coactivator 1 alpha (PGC-1α), and mitochondrial transcription factor A (TFAM), influencing the regulation and transcription of key mitochondrial proteins [81]. Our results indicate that exposure to SFN prevents the decrease in ΔΨm and the mitochondrial oxygen consumption rate, both affected by CdCl_2_ exposure (Figure 5). Additionally, pre-exposure to SFN before CdCl_2_ significantly decreases the level of mitochondrial ROS compared to the group exposed exclusively to CdCl2 (Figure 7B). Furthermore, a significant increase in mitochondrial mass was observed in the SFN+CdCl_2_ group (Figure 6). The beneficial effects on mitochondrial health that we observed suggest that exposure to SFN prevents CdCl_2_-induced mitochondrial dysfunction, and this could be primarily due to the increase in mitochondrial mass. Previous studies have suggested that SFN increases mitochondrial biogenesis in mice with accelerated senescence, which could explain the improvement in ETS activity, restoring ΔΨm [82]. It has also been demonstrated that SFN enhances multiple mitochondrial bioactivities and bioenergetic parameters such as ΔΨm, ATP, and ETS by promoting PGC-1α-dependent mitochondrial biogenesis and improving Nrf2-dependent mitochondrial redox regulation in HHL-5 cells and in rats fed a high-fat diet [63]. Additionally, among the mechanisms of compounds studied as potential strategies to protect against Cd damage is the restoration of mitochondrial mass, primarily by restoring mitochondrial biogenesis and regulating redox balance [80,83]. Furthermore, improvement in mitochondrial biogenesis has been associated with decreased ROS production and increased longevity [84]. Therefore, the results of our study suggest that SFN promotes mitochondrial restoration by increasing mitochondrial mass, which could contribute to its ability to mitigate Cd toxicity.

It is important to note that when evaluating intracellular ROS, no significant effect was observed in the SFN+CdCl_2_ group (Figure 7A). This lack of effect could be attributed to a masking phenomenon due to the increased mitochondrial mass. In other words, intracellular ROS might be slightly elevated in the SFN+CdCl_2_ group due to the increased mitochondrial mass observed in this study. Therefore, although there is a trend towards decreased intracellular ROS in this group, this effect did not reach statistical significance. This phenomenon was not observed in the assessment of mitochondrial ROS, as the fluorescence of MitoSOX^TM^ Red was corrected with MitoTracker^®^ Green fluorescence, which was used to measure mitochondrial mass to avoid this issue.

The specificity of probes for ROS detection could also be important in understanding the mechanisms of action of compounds, such as SFN, in protecting against heavy metal-induced oxidative stress like Cd. It has been observed that the H_2_DCFDA probe is more sensitive to hydrogen peroxide (H_2_O_2_), hydroxyl radicals (^•^OH), and peroxyl radicals (ROO), while the MitoSOX^TM^ Red probe primarily targets superoxide anion (O_2_^•−^) [30]. This differentiation is crucial since different ROS species may impact cellular and tissue health differently. In our study, the MitoSOXTM Red probe’s observation of a decrease in O_2_^•−^ levels in the SFN+CdCl_2_ group could suggest a protective effect of SFN, specifically, against this ROS species. The enzyme superoxide dismutase (SOD) is responsible for reducing O_2_^•−^ to H_2_O_2_. In the case of *C. elegans*, this organism possesses five SOD genes encoding two cytosolic CuZn-SOD enzymes (*sod-1* and *sod-5*), two mitochondrial Mn-SOD enzymes (*sod-2* and *sod-3*), and one extracellular CuZn-SOD enzyme (*sod-4*) [85]. It is known that Cd affects all isoforms. In Mn-SOD isoforms, Cd can replace the manganese ion, thereby reducing its activity, while in CuZn-SOD isoforms, momentary inhibition is attributed to a Cd/enzyme interaction [86]. Additionally, Cd directly damages the structure of the antioxidant enzymes catalase and glutathione peroxidase, which are responsible for reducing H_2_O_2_ to water [65,87]. SFN can eliminate both the O_2_^•−^ and H_2_O_2_ through a double hydrogen transfer without barriers or with low energy barriers, a process that requires the presence of Fe-SOD [88]. Additionally, it has been previously demonstrated that SFN increases the expression of antioxidant enzymes, either depending on the transcription factor Nrf2 or, in the case of *C. elegans*, through *skn-1c*, its orthologue [89,90]. Furthermore, in *C. elegans*, it has been observed that the transcription factor *daf-16* also activates key antioxidant enzymes [91,92]. It is important to note that in situations of excessive damage, such as a mutation in the *mev-1* gene, the protective potential of SFN is lost (Figure 7G), suggesting that the integrity of the succinate dehydrogenase enzyme is crucial in SFN’s protective effect against Cd toxicity. It has been reported that *the mev-1* gene plays an important role in regulating ROS and stress tolerance [93]. Mutation in the *mev-1* gene has been shown to increase Cd toxicity [94,95]. Genetic variation in *mev-1* is associated with increased in O_2_^•−^ levels and greater susceptibility to oxygen [66,67].

SFN acts as an indirect antioxidant, exerting its primary effect on oxidative stress and mitochondrial dysfunction through the activation of transcription factors such as *skn-1*/Nrf2 and *daf-16*/FoXO [47,96]. In *C. elegans*, both transcription factors belong to the pathway IIS. Therefore, the role of this pathway in SFN’s ability to protect against mitochondrial damage and Cd-induced toxicity has been investigated (Figure 8 and Figure 9). Firstly, we assessed the expression of DAF-2 and a mutant strain of the *age-1* gene. Both DAF-2 and AGE-1 are upstream regulators of SKN-1 and DAF-16, responsible for phosphorylating these transcription factors and preventing their translocation into the nucleus [31]. Our results indicate a decrease in DAF-2 expression in the SFN+CdCl_2_-treated group. Additionally, upon evaluating the survival of the *age-1* mutant strain, we found that SFN continued to protect against nematode mortality compared to the group exposed solely to CdCl_2_ (Figure 8). These findings suggest that *skn-1* and *daf-16* may not be phosphorylated and, therefore, may be activated. To support these hypotheses, we assessed survival in the QV225 (*skn-1c*) and GR1307 (*daf-16*) mutant strains, where we observed a decrease in the protection provided by SFN against Cd toxicity in the SFN+CdCl_2_ group compared to the WT strain. Furthermore, an increase in nuclear translocation of DAF-16 was observed in this group. These results indicate that the presence of these genes is essential for the protection conferred by SFN against Cd and that SFN may be activating them by regulating the IIS pathway. It has been previously reported that SFN extends lifespan and improves health in *C. elegans* by regulating IIS pathway signaling and activating the nuclear transcription factor DAF-16/FoxO [47]. Recent studies confirm our assumptions and show that various natural compounds use insulin signaling to enhance the oxidative stress response [97,98]. Notably, in mammals, SFN also regulates Nrf2 activation, the ortholog of the *skn-1c* gene. Mechanically, SFN binds to cysteine residues of Kelch-like ECH-associated protein 1 (Keap1), the inhibitor of Nrf2, inducing conformational changes that promote the release of “sequestered” Nrf2 and its subsequent translocation to the nucleus [99,100].

Additionally, the regulation of the IIS pathway by SFN, leading to the activation of *skn-1* and *daf-16*, would account for the observed protection against mitochondrial alterations. It has been observed that *skn-1c* is crucial for the expression of various genes related to mitochondrial biogenesis and mitochondrial health in *C. elegans*. It was discovered that skn-1 associates with proteins of the outer mitochondrial membrane [101]. The transcriptional activity of SKN-1 is essential for mitohormesis-mediated longevity and for maintaining mitochondrial homeostasis. Indeed, the reduction of skn-1 alters the morphology of the mitochondrial network, causes mitochondrial membrane depolarization, and increases cytoplasmic Ca^2+^ concentration [70]. Particularly, the reduction of SKN-1 decreases mitochondrial DNA content, highlighting its role in maintaining mitochondrial integrity [70].

Finally, another gene that plays a role in protection against Cd toxicity is *cdr-2*. Our findings revealed that the *cdr-2* gene might be implicated in the protection provided by SFN, as a decrease in SFN protection against CdCl_2_ was observed in the *cdr-2* mutant strain compared to the WT strain (Figure 4D). Ji-Yeon and colleagues demonstrated that the removal of *cdr-2* increased mortality and reduced reproductive potential in Cd-exposed *C. elegans* nematodes [56]. The *cdr* genes encode integral membrane proteins, primarily localized to the lysosomal membrane [60,102]. It has been postulated that alterations in the biological activity of *cdr* genes may trigger imbalances in fluids or disruptions in the osmoregulation of *C. elegans* [60,102]. Disruption in osmolarity has been shown to have various adverse effects on mitochondria, interfering with their normal function in energy production and other essential cellular processes. This includes affecting ΔΨm, ion transport, membrane permeability, and ATP generation, leading to reduced efficiency of cellular respiration and increased ROS production [103,104,105]. Despite sufficient evidence that Cd increases *cdr-2* expression as a protective mechanism, this study represents the first to establish a connection between SFN and *cdr-2* gene function, generating the hypothesis that cdr-2 may be relevant to the protection conferred by this antioxidant. Conducting additional transcriptomic and proteomic experiments would be crucial to validate these associations. Furthermore, it would be interesting to explore the impact of SFN on *cdr-1*, which is the most specifically Cd-sensitive gene within the cdr family.

**Figure 10:**
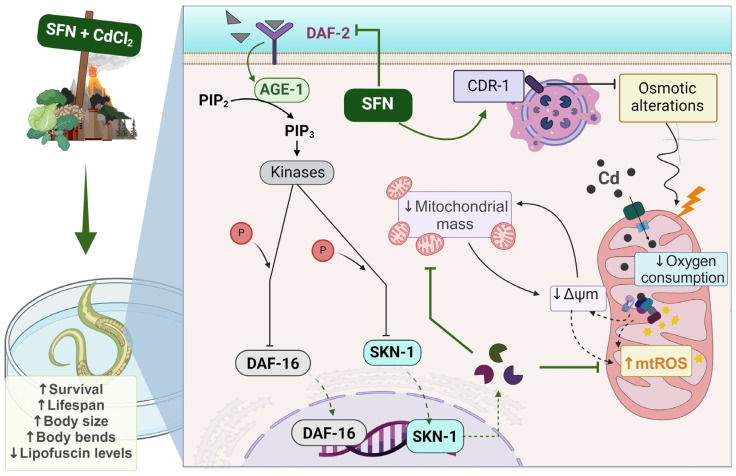
Integrative scheme. Cadmium (Cd) ingresses into the mitochondria and induces toxicity and alterations in mitochondrial function, such as the reduction of mitochondrial membrane potential (ΔΨm), mitochondrial oxygen consumption, and the increase of mitochondrial reactive oxygen species (ROS), which may be attributable to the reduction of mitochondrial mass. Conversely, sulforaphane (SFN) can prevent mitochondrial alterations by increasing mitochondrial mass through the regulation of the IIS pathway. Furthermore, the Cd response gene (CDR-1) could be implicated in Cd protection. Ultimately, the effect of SFN on mitochondrial alterations is evidenced by enhanced health in the nematode *Caenorhabditis elegans* (*C. elegans*). AGE-1: phosphatidylinositol 3-kinase gene protein, CdCl_2_: cadmium chloride, DAF-16: forkhead box O (FoxO) orthologous gene, DAF-2: receptor tyrosine kinase (IGFR) homologous gene protein, P: phosphate group, PDK-1: 3-phosphoinositide-dependent protein kinase 1, PIP3: phosphatidylinositol 3,4,5-trisphosphate, SKN-1c: ortholog of nuclear factor erythroid 2-related factor 2 (Nrf2). Created with biorender.com (published with permission from biorender.com).

As shown in the integrative scheme (Figure 10), our results suggest that exposure to Cd induces toxicity and alterations in mitochondrial function, such as decreased ΔΨm, mitochondrial oxygen consumption, and increased ROS levels, which may be attributable to reduced mitochondrial mass. On the other hand, SFN may prevent mitochondrial alterations by increasing mitochondrial mass through the regulation of the IIS pathway.

## 5. Conclusions

Exposure to SFN prior to CdCl_2_ exposure mitigates toxic effects and mitochondrial alterations, possibly by increasing mitochondrial mass, which may be related to the regulation of the IIS pathway. Additionally, the possibility arises that the genes *mev-1* and *cdr2* are important for the protection provided by SFN. These findings open up new possibilities for developing therapies to mitigate damage caused by Cd toxicity and oxidative stress in animals, highlighting mitochondria-targeted antioxidants as a promising tool.

## Figures and Tables

**Table 1:** Conditions of use of fluorescent probes.

Evaluation	Probe	Concentration (μM)	Exposure Time (Hours)
Intracellular ROS	2′,7′-dichlorodihydrofluoresceine diacetate (H_2_DCFDA)	50	4
Mitochondrial ROS	MitoSOX^TM^ Red	5	24
Mitochondrial mass	MitoTracker^®^ Green	5	24
Mitochondrial membrane potential (Δψm)	5,5,6,6-Tetrachloro-1,1,3,3-tetraethylbenzimidazolylcarbocyanine iodide (JC-1)	5	3

**Table 2:** Summary of lifespan data.

Treatment	Mean Lifespan (Days ± SEM)	Max Lifespan (Days)	*P (log-Rank)*		Number of Experiments (n)	Total Number of Nematodes
			vs. Control	vs. CdCl_2_		
Control	19 ± 0.45	25			5	109
DMSO	18 ± 0.50	25	ns		5	105
CdCl_2_	8 ± 0.56	20	<0.0001		5	104
SFN+CdCl_2_	14 ± 0.68	25	<0.0001	<0.0001	5	103
SFN	20 ± 0.58	30	0.0002		5	102

Mean lifespan values were calculated using the log-rank statistical test (Kaplan–Meier). All statistical evaluations were calculated using GraphPad Prism version 10. DMSO: dimethyl sulfoxide, CdCl_2_: cadmium chloride, SEM: standard error of the mean, SFN: sulforaphane, ns: Not significant.

## Data Availability

The data that support the findings of this study are available from the corresponding author upon reasonable request.

## Supplementary Materials

The following supporting information can be downloaded at: https://www.mdpi.com/article/10.3390/antiox13050584/s1, Figure S1: Calculation of the mean lethal concentration (LC50). Table S1: Characterization of *C. elegans* wildtype and mutant strains.

## Abbreviations

*age-1*	orthologous gene of phosphatidylinositol 3-kinase
*C. elegans*	*Caenorhabditis elegans*
Cd	cadmium
CdCl_2_	cadmium chloride
*cdr-1* o *2*	*cadmium response genes*
CI95	*confidence interval* 95%
*daf-16*	orthologous gene of forkhead box O (FoxO)
DAF-16	protein of the orthologous gene of forkhead box O (FoxO)
DAF-18	human tumor suppressor homologue/PTEN
*daf-2*	homolog of tyrosine kinase receptor (IGFR)
DAF-2	protein of gen homolog de tyrosine kinase receptor (IGFR)
DMSO	dimethyl sulfoxide
mtDNA	mitochondrial deoxyribonucleic acid
ER	Endoplasmic reticulum
ETS	electron transport system
FoxO	forkhead box O
FUdR	5-fluoro-2′-desoxiuridina
GFP	green fluorescent protein
H_2_DCFDA	6-chloromethyl-2′,7′-dichlorodihydrofluorescein diacetate
IC95	95% confidence interval
IIS	insulin/insulin-like growth factor signaling pathway
IGF-1	insulin-like growth factor-1
K_2_HPO_4_	potassium phosphate dibasic
KCl	potassium chloride
KH_2_PO_4_	potassium phosphate monobasic
LC_50_	Lethal concentration 50
MgSO_4_•7H_2_O	magnesium sulfate heptahydrate
*mtl-1 or 2*	Metallothionein-1 or 2
Na_2_HPO_4_	sodium hydrogen phosphate
NaCl	sodium chloride
NaClO	sodium hypochlorite
NaOH	sodium hydroxide
Nrf2	nuclear factor erythroid 2–related factor 2
PDK-1	3-phosphoinositide-dependent protein kinase 1
ROS	reactive oxygen species
SFN	sulforaphane
SGK-1	glucocorticoid-regulated kinase 1
*skn1a*	ortholog of Nrf1
*skn-1c*	ortholog of nuclear factor erythroid 2–related factor 2 (Nrf2)
ΔΨm	mitochondrial membrane potential
Sirt1	sirtuin 1
NRF1	nuclear respiratory factors 1
PGC-1α	peroxisome proliferator-activated receptor-gamma coactivator 1 alpha
TFAM	mitochondrial transcription factor A
VDAC	voltage-dependent anion channel
DMT1	divalent metal transporter 1
MCU	mitochondrial calcium uniporter
PIP_3_	phosphatidylinositol 3,4,5-triphosphate
JC-1	5,5,6,6′-tetrachloro-1,1′,3,3′ tetraethylbenzimi-dazoylcarbocyanine iodide
AKT1-2	protein kinases B
