# Supplementary material for: Sulforaphane Exposure Prevents Cadmium-Induced Toxicity and Mitochondrial Dysfunction in the Nematode Caenorhabditis elegans by Regulating the Insulin/Insulin-like Growth Factor Signaling (IIS) Pathway
Source: Antioxidants (Basel). 2024 May 9;13(5):584. doi: 10.3390/antiox13050584 (PMC11117759; doi:10.3390/antiox13050584)
Supplement: Supplementary file 1 [file antioxidants-13-00584-s001.zip › antioxidants-3003474-supplementary.pdf]

Supplementary material

Table S1. Characterization of *C. elegans* wildtype and mutant strains.

Strain name and WB ID	Genotype	Description	Ref
**N2**	*Wild type*	*C. elegans* was isolated in 1951 by W. L. Nicholas in Bristol from mushroom compost from a garden. Only a tiny part of the population is male; most are hermaphrodites. Its development has been described as lasting 3 days with a lifespan of 3 weeks. During their fertile stage, they can have an average of 300 eggs.	[32]
**VC128**	*mtl-2(gk125) V*	The metallothionein 2 enables Cd ion binding activity and zinc ion binding activity. Involved in response to Cd ion and response to heat. Mutant strain. T08G5.10. Superficially wild type. Made by Mark Edgley.	[33]
**RB1623**	*cdr-2(ok1996) V*	A gene involved in regulating protein deneddylation and stress response to cadmium ion. Predicted to be located in the cytoplasm. Mutant strain. C54D10.1. Homozygous. Outer Left sequence: GTTGGTGGCGTGAAGAATTT. Outer right Sequence: ATTCCGCTGCAAAATTAACG. Inner Left sequence: TGTCACTGGACAGCAACACA. Inner right sequence: AGCGTGTTCGCAAAGAGATT. Inner primer PCR Length: 2727 bp. Deletion Size: 1109 bp. Deletion left flank: ATTTTGGAATACCAATGCTTCTGACAAGAA. Deletion right flank: AAAAAAAATCAAGAAGAGTTTACAAATTTT. Made by OMRF Knockout Group.	[33]
**TK22**	*mev-1(kn1) III*	*mev-1* encodes the *C. elegans* ortholog for human mitochondrial succinate dehydrogenase cytochrome b560 subunit, the mutation of which is associated with a significantly decreased life span, increased reactive oxygen species, and severely impaired reproduction. Methylviologen (paraquat) sensitive. Made by Naoaki Ishii.	[34,35]
**TJ1052**	*age-1(hx546) II.*	Mutant strain. Long life. Normal fertility. Not temperature sensitive. Stress tolerant. Made by Hutchinson and Johnson.	[36]

**QV225**	*skn-1(zj15) IV*	SKN-1/Nrf2 proteins are members of the cap 'n' collar (CNC) family of transcription factors that are master regulators of oxidative stress resistance and longevity. skn-1(zj15) is a point mutation in an intron that causes mis-splicing of a fraction of mRNA and strongly reduces wildtype mRNA levels of the two long *skn-1a/c* variants. Hypomorphic allele of *skn-1* that may be propagated as a homozygote. High rate of embryonic lethality and slightly lower brood size compared to N2. Made by Keith Choe.	[37]
**GR2245**	*skn-1(mg570) IV*	skn-1(mg570) mutants lack SKN-1a but retain other SKN-1 isoforms. SKN-1a/Nrf1 is an unusual transcription factor associated with the endoplasmic reticulum via an N-terminal transmembrane domain. Mutant strain. Superficially wild type. Made by Nicholas Lehrbach.	[38,39]
**GR1307**	*daf-16(mgDf50)*I	The *daf-16* gene produces the sole *C. elegans* equivalent of the Forkhead box O (FOXO) transcription factor. Deficiency completely eliminates the *daf-16* coding region. Makes partial dauers on pheromone. Made by Shoshanna Gottlieb	[35]
**SJ4143**	*zcIs17* [ges-1::GFP(mit)]	Stable transgenic line expressing GFP in mitochondria of intestinal cells. Made by Cristina Benedetti.	[40,41]
**QQ202**	*daf-2(cv20[daf-2::GFP]) III*	Superficially wildtype. Made by Jeff Simske.	---
**OH16024**	*daf-16(ot971[daf-16: GFP]) I*	The CRISPR allele of *daf-16* is tagged at the C-terminus with GFP. Made by Ulkar Aghayeva.	[42]

Cd: cadmium, CNC: cap ’n’ collar, CRISPR: clustered regularly interspaced short palindromic repeats, FOXO4: forkhead box O4, GFP: green fluorescent protein, Mit: mitochondria, mRNA: messenger RNA, Mtl-2: metallothionein 2, Nrf2: nuclear factor erythroid 2–related factor 2, PCR: polymerase chain reaction.

a) Graphpad prism 5

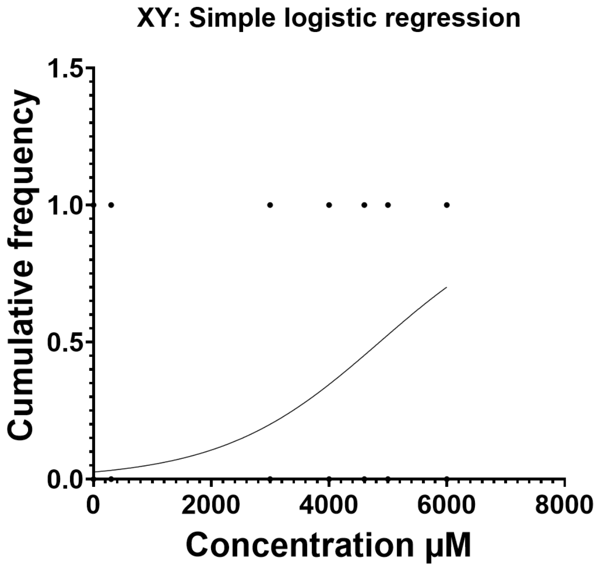

Std. Error	
X at 50%	79.14
95% Confidence Intervals	
X at 50%	4707 to 5019
Goodness of Fit	
Tjur's R squared	43
Cox-Snell's R squared	0.8208
Model deviance, G squared	3966
Data summary	
Rows in table	1960
Rows skipped (missing data)	0
Rows analyzed (#observations)	1960
Number of 1	642
Number of 0	1318
Number of parameter estimates	2
LC_{50}= 4 858 **μM**	

b) Probit analysis

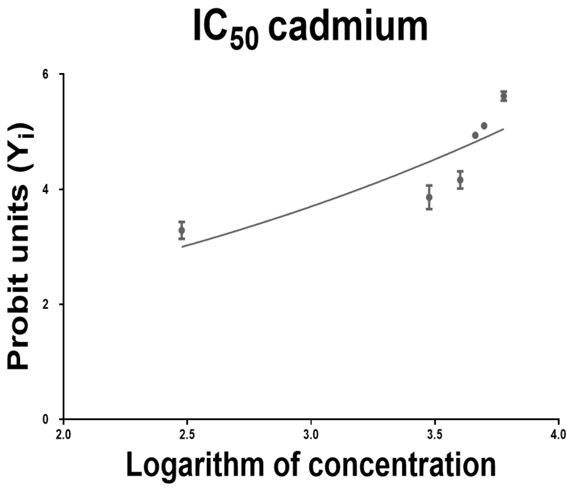

R^2	0.7204	
Slope	6.1334	
Intercept	-17.61	
Lethal concentration 16	3344.333	μm
Lethal concentration 84	7054.016	μm
Standard deviation (S)	18.54.841	
Population size (Yi= 0.07-0.93) N´	1377	
Standard error of measurement (SEM)	70.689	μM
t(a=0.05, gl=N-2)	1.96	
to.o5*EEM	138.5514	
Lower Control Limit (LCL)	4719	μM
Upper Control Limit (UCL)	4996	μM
**LC_{50}= 4 857 ±** 138.6 **μM**		

Figure S1: Calculation of the mean lethal concentration (LC_{50}). A) LC_{50} was calculated using the GraphPad prism 10, and B) LC50 was calculated using Probit analysis.
